# Adiposity mediates the association between whole grain consumption, glucose homeostasis and insulin resistance: findings from the US NHANES

**DOI:** 10.1186/s12944-018-0805-6

**Published:** 2018-09-17

**Authors:** Mohsen Mazidi, Niki Katsiki, Andre Pascal Kengne, Dimitri P. Mikhailidis, Maciej Banach

**Affiliations:** 1 Key State Laboratory of Molecular Developmental Biology,Institute of Genetics and Developmental Biology, Chinese Academy of Sciences, Beijing, 100101 People’s Republic of China; 2 Second Propedeutic Department of Internal Medicine, Medical School, Aristotle University of Thessaloniki, Hippokration Hospital, Thessaloniki, Greece; 3 Non-Communicable Disease Research Unit, South African Medical Research Council and University of Cape Town, Cape Town, South Africa; 4 Department of Clinical Biochemistry, Royal Free Campus, University College London Medical School, University College London (UCL), London, UK; 5 Department of Hypertension, Chair of Nephrology and Hypertension, Medical University of Lodz, Lodz, Poland; 6 Polish Mother’s Memorial Hospital Research Institute (PMMHRI), Lodz, Poland; 7 Cardiovascular Research Centre, University of Zielona Gora, Zielona Gora, Poland

**Keywords:** Whole grain, Insulin resistance, Inflammation, Adiposity

## Abstract

**Background:**

Growing evidence suggests an inverse association between whole grain (WG) consumption and insulin resistance (IR) or inflammation. However, it is still unclear whether adiposity plays a role in this relationship.

We investigated whether the associations between WG intake with IR, glucose homeostasis and inflammation are mediated by adiposity in US adults.

**Methods:**

The 2005–2010 National Health and Nutrition Examination Surveys participants were included. WG intake was assessed and markers of IR and glucose homeostasis, inflammation, general and central adiposity. Analysis of co-variance and mediation analysis were applied, while accounting for survey design.

**Results:**

Overall 16,621 participants were included in this analysis (mean age = 47.1 years, 48.3% men). After adjustment for age, gender, and race, mean C-reactive protein (CRP), apolipoprotein B (apo-B), fasting blood glucose (FBG), insulin, homeostatic model assessment of IR (HOMA-IR) and β cell function (HOMA-β), hemoglobin A_1c_ (HbA_1c_), and 2 h glucose after an oral glucose tolerance test decreased with increasing quarters of WG (all *p* < 0.001). Body mass index (BMI) had significant mediation effects on the associations between WG intake and CRP, apo-B, fasting glucose, insulin, HOMA-IR, HOMA-B, HbA_1c_, triglyceride to high density lipoprotein-cholesterol (TG:HDL-C) ratio and triglyceride-glucose (TyG) index (all *p* < 0.05) after adjustment for age, gender, race/ethnicity, educational status, smoking and level of physical activity. Both waist circumference (WC) and anthropometrically predicted visceral adipose tissue (apVAT) mediated the association between WG intakes with CRP, FBG, HbA_1c_, TG:HDL-C ratio and TyG index, i.e. WC and apVAT had indirect effect (all *p* < 0.05).

**Conclusion:**

Our findings provide insights into the favourable impact of WG consumption on IR and inflammation, which may be affected by both central and visceral adiposity, i.e. the link between WG with IR and inflammation is more mediated in overweight/obese compared with lean individuals.

## Background

Diet is an important lifestyle component involved in the development of chronic diseases such as cardiovascular (CV) disease (CVD) and diabetes mellitus (DM). Studies have suggested that a high consumption of whole-grain (WG) foods is associated with a lower risk of CVD [1, 2] and DM [3]. In general however, the underlying mechanisms of these associations have not been completely elucidated, but could involve adiposity. This has been supported by long-term data showing a reduction of postprandial insulin and glucose responses after 2–4 weeks of WG rye or wheat diet in overweight men [4, 5]. Further studies reported that WG intake reduced the risk of coronary heart disease (CHD), CVD and cancer, as well as all-cause death and mortality from respiratory diseases, infectious diseases, DM, and other non-CV and non-cancer causes [5]. However, randomized control trials (RCTs) found no significant effect of WG foods on major CVD risk factors [6–8]. In this context, a recent systematic review and meta-analysis of 9 RCTs indicated that there is insufficient evidence on the effect of WG diets on CV outcomes or major CVD risk factors [8].

Mediation analysis could clarify the role of adiposity, if any, in the relation between WG intake and cardiometabolic risk [9]. From a statistical standpoint, mediation analysis can be used to explore and quantify the extent to which the relationship between an exposure and an outcome of interest occurs through the effect of a third variable. The traditional approach to mediation analysis tends to produce a bias when there is a confounding uncontrolled mediator outcome or an interaction between exposure and mediator. Using the counterfactual framework in mediation analysis, unbiased valid estimates of direct and indirect effects can be obtained [9, 10].

The present study aimed to evaluate the associations between WG consumption and markers of glucose/insulin homeostasis and inflammation, as well as to assess the mediation effect of adiposity parameters on these relationships using “mediation analysis”. These analyses were applied to a large, representative sample of US adults.

## Methods

### Population characteristics

The National Health and Nutrition Examination Survey (NHANES) is a series of ongoing repeated cross-sectional surveys conducted by the US National Center for Health Statistics (NCHS) [11]. The NCHS Research Ethics Review Board approved the NHANES protocol and consent was obtained from all participants [12]. The current study was based on analysis of data collected from 2005 to 2010. Data collection on demographics occurs through in-home administered questionnaires, while anthropometric, inflammation and biochemistry data were collected by trained personnel using mobile exam centers. More detailed information is available elsewhere [13, 14]. Data on level of education were collected as self-report to this question “What is the highest grade or level of school completed or the highest degree received?”

Glycated hemoglobin (HbA_1c_) was measured using a Tosoh A1C 2.2 Plus Glycohemoglobin Analyzer (San Francisco, USA). Plasma glucose [fasting and 2 h post oral glucose tolerance test (OGTT)] was measured by a hexokinase method using a Roche/Hitachi 911 Analyzer and Roche Modular P Chemistry Analyzer (Organon Technika, Durham, NC). Other biological tests details are available in the NHANES Laboratory/Medical Technologists Procedures Manual [15]. C-reactive protein (CRP) levels were measured by latex-enhanced nephelometry [14]. Homeostatic model assessment of insulin resistance (HOMA-IR) and β-cell function (HOMA-B) were calculated as follows: HOMA-IR = [fasting glucose-FBG (nmol/L) * fasting insulin (mU/mL)/22.5], and HOMA-B = [20 × fasting insulin (μU/ml)]/ [FBG (mmol/l) − 3·5] [16]. Triglyceride-glucose (TyG) index was calculated as ln[fasting triglyceride;TG (mg/dl) × FBG (mg/dl)/2] [16]. The anthropometrically predicted visceral adipose tissue (apVAT) was calculated with gender-specific validated equations: for men: 6 *waist circumference (WC) – 4.41 * proximal thigh circumference + 1.19 * age – 213.65; for women: 2.15 * WC – 3.63 * proximal thigh + 1.46 * age + 6:22 * body mass index (BMI) -92.713 for women [17].

### WG consumption

Dietary intake was assessed via 24 h recall obtained by a trained interviewer during the mobile examination center visit, with the use of a computer-assisted dietary interview system with standardized probes, i.e. the US Department of Agriculture Automated Multiple-Pass Method (AMPM) [18]. Briefly, the type and quantity of all foods and beverages consumed in a single 24 h period before the dietary interview (from midnight to midnight) were collected with the use of AMPM. AMPM is designed to enhance complete and accurate data collection while reducing respondent burden [18, 19]. Detailed descriptions of the dietary interview methods are provided in the NHANES Dietary Interviewer’s Training Manual [20]. The MyPyramid Equivalents Database for USDA Survey Food Codes was used to calculate WG intake [20].

### Statistical analysis

Data analyses followed the Centers for Disease Control and Prevention (CDC) guidelines for analysis of complex NHANES data, accounted for the masked variance and used the recommended weighting methodology [21]. All analyses were conducted with the use of SPSS version 22.0 (IBM Corp, Armonk, NY). The Kolmogorov-Smirnov test was used to evaluate the normal distribution of variables. We computed age, race, and gender-adjusted mean of insulin resistance (IR) or inflammatory markers across the quarters of WG consumption, using analysis of covariance (ANCOVA). Multi-collinearity for the multiple linear regressions was assessed with variance inflation factors (VIF) at each step [22]. Multi-collinearity was considered high when the VIF was > 10 [22].

In the present study, we assessed the total, direct, and indirect effects of WG intake on IR or inflammation with BMI, WC or apVAT as a mediator by using the counterfactual framework [23, 24]. This method has been fully explained elsewhere [25, 26]. In this approach, total effect can be divided into direct effect (not mediated by BMI, WC or apVAT) and indirect effect (mediated by BMI, WC or apVAT). The SPSS Macro developed by Preacher and Hayes [26] was used to evaluate the direct and indirect effect of WG intake on IR or inflammation with BMI, WC or apVAT as mediators.

## Results

### General characteristics

A total of 16,621 participants met the criteria for inclusion in the current analysis. Their characteristics are summarized in Table 1. Overall, mean age was 47.1 years and 48.3% of the participants were men. Non-Hispanic white (69.4%) was the largest racial group and other Hispanic (4.5%) the smallest racial group. Furthermore, 56.1% of the participants were married, while 56.4% had achieved more than high school (Table 1). Mean and standard error of mean (SEM) of BMI, WC and apVAT were 28.7 ± 0.05 (kg/m^2^), 98 ± 0.1 (cm) and 179.2 ± 1.2, respectively (Table 1).

**Table 1:** Demographic and clinical characters of subjects

Characteristics		Overall	*P*-value
Sex	Men (%)	48.3%	< 0.001
	Women (%)	51.7%	
Age (Years)		47.1 ± 1.1	
Race/Ethnicity	White (non-Hispanic) (%)	69.4%	< 0.001
	Non-Hispanic Black (%)	11.5%	
	Mexican-American (%)	8.4%	
	Other Hispanic (%)	4.5%	
	Other (%)	6.2%	
Marital Status	Married (%)	56.1%	< 0.001
	Widowed (%)	61.1%	
	Divorced (%)	10.1%	
	Never married (%)	17.9%	
Education Status	Less than high school (%)	19.1%	< 0.001
	Completed high school (%)	24.4%	
	More than high school (%)	56.4%	
Body mass index (kg/m2)		28.7 ± 0.05	
Waist circumference (cm)		98.2 ± 0.12	
apVAT		179.2 ± 1.18	
TyG index		8.78 ± 0.002	
Serum Hs-CRP (mg/dl)		0.43 ± 0.001	
Serum Apolipoprotein (B) (mg/dL)		94.2 ± 0.25	
Fasting blood glucose (mg/dl)		100.2 ± 0.021	
Plasma Insulin (uU/mL)		2.31 ± 0.008	
HOMA_IR		0.89 ± 0.008	
HOMA_B		4.78 ± 0.002	
HbA1c (%)		5.66 ± 0.004	
2-h blood glucose(mg/dL)		120.3 ± 0.98	

Value expressed as a mean and SEM or percent. Abbreviation = Abbreviations: HOMA_IR, Homeostatic model assessment of insulin resistance; HOMA_B, Homeostatic model assessment of β-cell function, TyG index, triglyceride-glucose index Hs-CRP; high senility C-reactive protein,; HbA1c: Glycated haemoglobin, apVAT: anthropometrically predicted visceral adipose tissue

Age, gender and race-adjusted mean of markers of IR and inflammation (i.e. CRP, apolipoprotein B (apo-B), FBG, insulin, HOMA-IR, HOMA-B, HbA_1c_, 2 h glucose and TG:high density lipoprotein-cholesterol (HDL-C) ratio were significantly decreased across increasing quarters of WG consumption (*p* < 0.001 for all comparisons). In contrast, there were no significant differences in TyG index across quarters of WG consumption (*p* = 0.082, Table 2).

**Table 2:** Age-, sex-, and race-adjusted mean of markers of insulin resistance and inflammation across quartiles of WG consumption

Variables	Quarters of WG consumption				*p* –value ^a^
	1	2	3	4	
n	4153	4158	4166	4144	
Serum Hs-CRP (mg/dl)	0.45 ± 0.01	0.42 ± 0.02	0.38 ± 0.02	0.33 ± 0.01	< 0.001
Serum Apolipoprotein (B) (mg/dL)	95.6 ± 1.45	94.1 ± 1.95	93.9 ± 2.72	87.4 ± 1.12	< 0.001
Fasting blood glucose (mg/dl)	105.3 ± 1.63	102.6 ± 1.26	101.2 ± 1.82	98.5 ± 2.83	< 0.001
Plasma Insulin (uU/mL)	2.45 ± 0.02	2.41 ± 0.03	2.40 ± 0.02	2.36 ± 0.02	< 0.001
HOMA_IR	0.98 ± 0.03	0.95 ± 0.02	0.91 ± 0.04	0.88 ± 0.02	< 0.001
HOMA_B	5.21 ± 0.02	5.08 ± 0.03	4.63 ± 0.01	4.04 ± 0.03	< 0.001
HbA1c (%)	5.86 ± 0.03	5.77 ± 0.06	5.71 ± 0.01	5.53 ± 0.02	< 0.001
2-h blood glucose(mg/dL)	124.6 ± 1.4	122.8 ± 1.9	119.2 ± 2.6	116.1 ± 1.7	< 0.001
TyG index	8.89 ± 0.02	8.88 ± 0.03	8.12 ± 0.01	8.01 ± 0.02	0.082

Abbreviations: HOMA_IR, Homeostatic model assessment of insulin resistance; HOMA_B, Homeostatic model assessment of β-cell function, TyG index, triglyceride-glucose index Hs-CRP; high senility C-reactive protein; HbA1c Glycated haemoglobin. Values expressed as estimated mean and standard error

^a^ *p*-values for linear trend across quartiles of hs-CRP. Variables were compared across quartiles of CRP using analysis of c-variance (ANCOVA) test

### Association between WG intake, BMI, WC, apVAT and markers of IR and inflammation

With regard to the action theory [i.e. the examination of the link between WG and hypothesized mediators (BMI, WC, apVAT), after multivariable adjustment], there was a significant association between BMI (β: − 0.255, *p* < 0.001), WC (− 0.446, *p* < 0.001), apVAT (− 1.620, *p* = 0.036) and WG intake (Table 2). When the “total effect” was calculated by examining the association between WG intake and markers of IR or inflammation in multivariable models without adjusting for potential mediators, with the exception of apo-B and HOMA-B, markers of IR or inflammation were negatively and significantly associated with WG intake (*p* < 0.001 for all comparisons, Table 3). When the ‘conceptual theory’ was tested [i.e. the association between mediators (BMI, WC and apVAT) and markers of IR or inflammation], all potential mediators had significant and positive associations with markers of IR or inflammation (*p* < 0.001 for all comparisons 1, Table 4).

**Table 3:** Estimates of regression coefficients (95% CIs) for the association between the whole grain intake, BMI, WC, apVAT (action theory), and markers of insulin resistance and inflammation (total effect) among US adults in NHANES

Mediator	Estimate	95% CI	P
BMI	−0.255	(− 0.344 to − 0.165)	< 0.001
WC	− 0.446	(− 0.655 to − 0.233)	< 0.001
apVAT	−1.620	(−3.136 to − 0.107)	−0.036
*Outcome*			
Serum Hs-CRP (mg/dl)	−0.029	(− 0.040 to − 0.018)	< 0.001
Serum Apolipoprotein (B) (mg/dL)	− 0.391	(− 0.881 to 0.133)	0.121
Fasting blood glucose (mg/dl)	−1.049	(− 1.54 to − 0.55)	< 0.001
Plasma Insulin (uU/mL)	− 0.023	(− 0.038 to − 0.009)	< 0.001
HOMA_IR	− 0.031	(− 0.047 to − 0.016)	< 0.001
HOMA_B	− 0.006	(− 0.021to 0.009)	0.425
HbA1c (%)	−0.025	(−0.039 to − 0.012)	< 0.001
2-h blood glucose(mg/dL)	−2.431	(−3.521to − 1.34)	< 0.001
TyG index	− 0.014	(− 0.023 to − 0.005)	< 0.001

*Abbreviations:* BMI body mass index, WC waist circumference, apVAT Anthropometrically-predicted visceral adipose tissue, HOMA_IR, Homeostatic model assessment of insulin resistance; HOMA_B, Homeostatic model assessment of β-cell function HOMA_S; Homeostatic model assessment of insulin sensitivity, TyG index, triglyceride-glucose index Hs-CRP; high senility C-reactive protein, HbA1c Glycated haemoglobin. All estimates were adjusted for age, sex, race/ethnicity, educational, smoking and level of physical activity. Estimates for mediator and outcomes correspond to the regression coefficients α and £, respectively, in Fig. 1

**Table 4:** Estimates of regression coefficients (95% CIs) for the association between BMI, WC, apVAT and markers of insulin resistance and inflammation (conceptual theory) among US adults

Outcomes	BMI			WC			apVAT		
	Estimate	95% CI	P	Estimate	95% CI	P	Estimate	95% CI	P
Serum Hs-CRP (mg/dl)	0.028	(0.026 to 0.029)	< 0.001	0.012	(0.011 to 0.012)	< 0.001	0.003	(0.002 to 0.003)	< 0.001
Serum Apolipoprotein (B) (mg/dL)	0.523	(0.440 to 0.607)	< 0.001	0.289	(0.253 to 0.326)	< 0.001	0.086	(0.068 to 0.104)	< 0.001
Fasting blood glucose (mg/dl)	0.876	(0.792 to 0.951)	< 0.001	0.402	(0.367 to 0.437)	< 0.001	0.088	(0.073 to 0.104)	< 0.001
Plasma Insulin (uU/mL)	0.058	(0.056 to 0.060)	< 0.001	0.026	(0.026 to 0.027)	< 0.001	0.007	(0.006 to 0.007)	< 0.001
HOMA_IR	0.066	(0.064 to 0.068)	< 0.001	0.030	(0.029to 0.031)	< 0.001	0.007	(0.006 to 0.007)	< 0.001
HOMA_B	0.037	(0.034 to 0.039)	< 0.001	0.017	(0.016to 0.018)	< 0.001	0.005	(0.004 to 0.005)	< 0.001
HbA1c (%)	0.029	(0.027 to 0.031)	< 0.001	0.012	(0.011 to0.013)	< 0.001	0.003	(0.002 to 0.003)	< 0.001
2-h blood glucose(mg/dL)	1.532	(1.352 to 1.723)	< 0.001	0.709	(0.621 to0.769)	< 0.001	0.211	(0.175 to 0.246)	< 0.001
TyG index	0.029	(0.027 to 0.030)	< 0.001	0.015	(0.014 to 0.015)	< 0.001	0.004	(0.004 to 0.004)	< 0.001

*Abbreviations*: BMI: body mass index, WC, waist circumference, apVAT, Anthropometrically-predicted visceral adipose tissue, HOMA_IR, Homeostatic model assessment of insulin resistance; HOMA_B, Homeostatic model assessment of β-cell function HOMA_S; Homeostatic model assessment of insulin sensitivity, TyG index, triglyceride-glucose index, Hs-CRP; high senility C-reactive protein. All estimates were adjusted for age, sex, race/ethnicity, educational, smoking and level of physical activity*.* Regression coefficient β is shown in Fig. 1

### Direct and indirect effects of WG intake on markers of IR and inflammation with BMI, WC and apVAT as mediators

Table 5 shows the direct effect, indirect effect, proportion of mediation effect, and Sobel statistics for testing indirect effects. For BMI, the mediated effects (indirect effect) were significant for the associations between WG intake and CRP, apo-B, FBG, plasma insulin, HOMA-IR, HOMA-B, HbA_1c_, TG;HDL-C ratio and TyG index (*p* < 0.05 for all comparisons), but not for 2 h glucose. Furthermore, WC and apVAT mediated the association between WG intake and markers of IR and inflammation, including CRP, FBG, HbA_1c_ and TyG index (*p* < 0.05 for all comparisons).

**Table 5:** Direct and indirect effects of whole grain consumption on markers of insulin resistance and inflammation with BMI, WC and apVAT as mediators among US adults

Mediator and outcomes	Direct effect (£^,^)		Indirect effect (α#β)3		Proportion of mediation, %
	Estimate	P	Estimate	Sobel test statistic	
BMI					
Serum Hs-CRP (mg/dl)	−0.020	< 0.001	− 0.007	< 0.001	26.6%
Serum Apolipoprotein (B) (mg/dL)	−0.382	0.124	−0.083	< 0.001	17.6%
Fasting blood glucose (mg/dl)	−0.832	< 0.001	−0.235	< 0.001	22.2%
Plasma Insulin (uU/mL)	−0.014	0.019	−0.0096	0.013	40.1%
HOMA_IR	−0.021	< 0.001	−0.010	0.014	33.7%
HOMA_B	−0.001	0.882	−0.005	0.021	84.3%
HbA1c (%)	−0.019	< 0.001	−0.078	< 0.001	28.3%
2-h blood glucose(mg/dL)	−2.235	< 0.001	− 0.198	0.0831	8.1%
TyG index	−0.007	0.102	−0.007	< 0.001	51.3%
WC					
Serum Hs-CRP (mg/dl)	−0.021	< 0.001	−0.058	< 0.001	21.5%
Serum Apolipoprotein (B) (mg/dL)	−0.307	0.214	−0.079	0.074	20.4%
Fasting blood glucose (mg/dl)	−0.853	< 0.001	−0.198	< 0.001	18.8%
Plasma Insulin (uU/mL)	−0.014	< 0.001	−0.075	0.062	34.2%
HOMA_IR	−0.021	< 0.001	−0.008	0.067	28.3%
HOMA_B	−0.001	0.846	−0.004	0.083	77.1%
HbA1c (%)	−0.020	< 0.001	−0.006	< 0.001	23.1%
2-h blood glucose(mg/dL)	−2.196	< 0.001	− 0.204	0.092	8.5%
TyG index	−0.006	0.147	−0.007	< 0.001	52.1%
apVAT					
Serum Hs-CRP (mg/dl)	0.003	0.733	−0.005	0.022	62.3%
Serum Apolipoprotein (B) (mg/dL)	0.351	0.462	−0.062	0.522	1.2%
Fasting blood glucose (mg/dl)	−0.471	0.273	0.163	0.026	25.3%
Plasma Insulin (uU/mL)	−0.006	0.561	−0.005	0.488	44.5%
HOMA_IR	−0.009	0.455	−0.005	0.509	37.2%
HOMA_B	0.001	0.952	−0.002	0.644	99.2%
HbA1c (%)	−0.020	0.082	−0.005	0.017	21.1%
2-h blood glucose(mg/dL)	−1.162	0.244	−0.410	0.154	26.0%
TyG index	−0.005	0.511	−0.007	0.023	57.2

*Abbreviations:* BMI: body mass index, WC, waist circumference, apVAT, Anthropometrically-predicted visceral adipose tissue, HOMA_IR, Homeostatic model assessment of insulin resistance; HOMA_B, Homeostatic model assessment of β-cell function HOMA_S; Homeostatic model assessment of insulin sensitivity, TyG index, triglyceride-glucose index Hs-CRP; high senility C-reactive protein. All estimates were adjusted for age, sex, race/ethnicity, educational, smoking and level of physical activity. Regression coefficients α, β, and £^,^ are shown in Fig. 1

Direct effects showed that WG was associated with CRP, FBG, plasma insulin, HOMA-IR, HbA_1c_ and 2 h glucose, even after adjustment for BMI or WC. In contrast, for apVAT, the estimates of direct effects were not significant. The highest mediated effects by BMI, WC and apVAT were those for HOMA-B (84.3%), TyG index (52.1%) and CRP (62.3%), respectively.

## Discussion

**Fig. 1:**
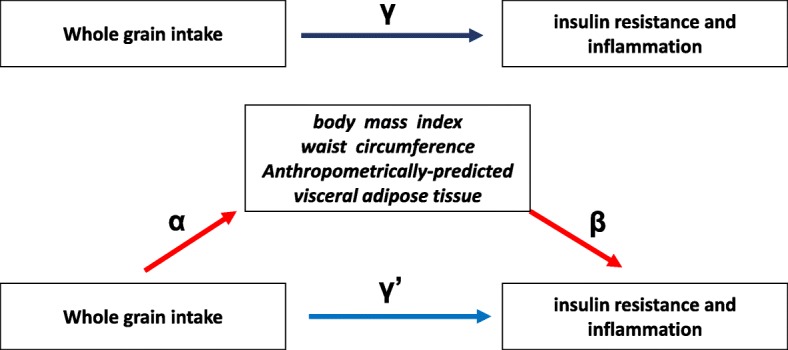
Mediation model for the association between whole grain (WG) intake, insulin resistance (IR) and inflammation with body mass index (BMI), waist circumference (WC) and anthropometrically-predicted visceral adipose tissue (apVAT) as mediators. Path α represents the regression coefficient for the association of WG intake with BMI, WC and apVAT. Path β represents the regression coefficient for the association of BMI, WC and apVAT with IR and inflammation. The product of regression coefficients α and β represents the mediated effect (indirect effect) of BMI, WC or apVAT (α#β). Path £’ represents the direct effect of WG intake with IR and inflammation, after adjustment for BMI, WC or apVAT. Path £ represents the simple total effect of WG intake on IR and inflammation, without adjustment for BMI, WC or apVAT

The mediation analysis conducted in the current study supports a relationship between WG intake and markers of IR and inflammation, being, at least partially, mediated by the effect of both general and central adiposity (Fig. 1). Altogether, our findings suggest some pathways through which WG consumption may affect the long-term risk of chronic diseases, including CVD and DM.

Our findings are in line with previous observational and interventional studies that demonstrated an inverse association between WG consumption and inflammatory markers [27–29]. In contrast, there are also studies showing no effects of WG on CRP or interleukin (IL)-6 [7, 30]; low statistical power may, at least in part, explain these discripancies. Several mechanisms have been proposed in relation to the health benefits induced by WG. It has been reported that WG are a rich source of fiber, minerals [magnesium (Mg), potassium, phosphorous, selenium, manganese, zinc, and iron], vitamins (especially high in vitamins B and E), phenolic compounds, phytoestrogens (lignans) and related antioxidants [31]. These compounds have been found to favorably affect markers of inflammation and glucose homeostasis [32, 33]. Dietary fibers in WG can also exert their anti-inflammatory action by decreasing lipid oxidation, which in turn is associated with reduced inflammation [34].

In line with our findings, in two reports from the Framingham Offspring Cohort Study, the number of daily servings of WG foods was inversely related with fasting insulin concentrations and IR, but not HbA_1c_ [35, 36]. In the Insulin Resistance Atherosclerosis Study, a greater intake of dark bread and cereals were associated with higher insulin sensitivity [37]. A similar association between WG intake and insulin sensitivity was observed in a cross-sectional study of 285 adolescents from Minnesota [38]. In these studies, the associations were more pronounced in overweight participants [36, 38].

In contrast, a smaller study carried out in Finland did not find a significant difference in incident type 2 DM (T2DM) when comparing consumers of WG foods above and below the extreme quartiles of intake, although there was a trend suggesting a reduced risk of developing T2DM among greater consumers of WG foods [39]. Liu et al. found that the risk of T2DM increased across quintiles of refined: WG food intake ratio. In detail, after adjustment for age and energy intake, women in the highest quintile of refined WG: food intake had a 57% greater risk of incident T2DM compared with women in the lowest intake ratio [40]. Pereira et al. compared insulin sensitivity in overweight T2DM patients on diets that incorporated refined or WG; a greater rate of glucose infusion was achieved during an euglycemic hyperinsulinemic clamp test in those patients on WG diet [41].

With regard to the beneficial impact of WG on glucose and insulin metabolism, a number of possible mechanisms have been proposed. WG foods are known to delay digestion and absorption of carbohydrates. Food structure has been found to be more important than gelatinization or the presence of viscous dietary fiber in determining glycemic response [42]. Refining grains tends to increase the glycemic response, whereas WG tend to slow it [43]. Other properties of WG, which make them beneficial for health and glucose/insulin balance, could be their relatively high antioxidant activity [44]. Soluble antioxidants include phenolic acids, flavonoids, tocopherols and avenanthramides in oats [44]. Studies suggested that T2DM may be reduced by dietary antioxidants intake [39, 45]. Grain lipids comprise about 75 g unsaturated fatty acids/100 g, of which there are approximately equal amounts of oleic and linoleic acid and 1–2 g linolenic acid/100 g. Therefore, WG consist of a high load of unsaturated fatty acids which can reduce the risk of T2DM [46]. Furthermore, WG also contain phytic acid, lectins, phenolics, amylase inhibitors and saponins which have also been shown to lower plasma glucose and insulin [47].

It has been reported that dietary fiber could be affected by macrobiota (fermentation) in the colon [48] and produce short chain fatty acids (SCFA), including acetate, butyrate and propionate [49]. Several mechanisms linking SCFA to insulin sensitivity and T2DM have been proposed, including the inter-organ effects on adipose tissue function and lipid storage capacity, inflammatory profile, as well as liver and skeletal muscle substrate metabolism [50–52].

Another finding of the present analysis was that adiposity parameters could, at least partially, mediate the associations between WG and IR or inflammatory markers. Observational studies have also shown an inverse relationship between WG intake and BMI, WC and risk of weight gain [53]. In particular, central adiposity, has been strongly associated with plasma inflammatory protein concentrations, and visceral adipose tissue is known to secrete a number of pro-inflammatory adipokines, including tumor necrosis factor a, IL-6 and Plasminogen activator inhibitor-1 (PAI-1) [53]. Therefore, it is possible that WG intake could be related to lower inflammatory protein levels by preventing weight increase, promoting weight maintenance, and reducing visceral adiposity. In another study*,* the authors applied the same techniques on cross-sectional data from 4700 adults aged 20–90 years, and reported that reducing abdominal obesity might play an important role in the pathway through which Mediterranean diet consumption reduces insulin resistance and inflammation [54]. With regard to the role of adiposity on the association between CRP and WG consumption, the 4 studies that evaluated the strength of the association between dietary patterns and CRP before and after adjustments for indices of adiposity, have shown a weakening of the association for 3 studies [55–57] and a strengthening for 1 [58]. A weakening association following the adjustment could be consistent with at least part of the effects of the dietary pattern being mediated through changes in adiposity and/or adipose tissue metabolism. In the present study, we found that link between WG with IR and inflammation is more mediated in overweight/obese individuals compared with lean. These findings support the implementation of WG intake in both overweight-obese individuals (who usually also have increased IR and inflammation) and normal-weight individuals to minimize the development/progression of these metabolic disorders.

The current study has major strengths, including the use of cause mediation to investigate the effects of general and central adiposity on the relationship of WG intake with IR and inflammation. The large sample size afforded adequate statistical power to conduct the complex analysis with little risk of multiple comparisons effects. The quality of metabolic and anthropometric measurements obtained at the NHANES study visit, including WC measurement as a marker of visceral adiposity and a glucose tolerance test as a measure of insulin sensitivity, allowed for the adjustment of these variables with a high degree of precision, potentially providing insight to the pathway by which WG consumption could influence inflammatory markers and insulin/glucose homeostasis.

Our analysis also has limitations. The cross-sectional nature precludes any reliable establishment of the sequence of events between WG intake, change in adiposity and IR/inflammation. It is however, less an expectation that IR or inflammation can cause individuals not to consume WG, while the reciprocal relationship between adiposity and IR/inflammation has been largely documented. The mediated effect of WC may be affected by BMI, or vice versa, because of the high correlation between WC and BMI. In the usual databases, this could have been addressed by considering 2 mediators (BMI and WC) simultaneously in a model [45]. However, this was not feasible in the conventional or causal mediation models with the use of the complex survey design as in the present study. To address this point, we used other adiposity markers, i.e. apVAT. Although BMI and WC are commonly used to estimate obesity, these markers may be inaccurate and can lead to bias in measuring adiposity. Therefore, the association between WG consumption and overall adiposity can be underestimated when BMI is used as a marker of adiposity. Moreover, because of a high chance of co-linearity, we excluded potential confounding factors, which could have increased the chance of residual bias in our models. Lastly, we need to mention that, due to the nature of our study (cross-sectional), reverse causality for the determination of the cause and effect (direction of causality) could be present. In this regard, RCTs are warranted to confirm our results.

The current study has significant clinical and public health implications, regarding the role of adiposity in the association between WG consumption, IR and inflammation markers. This is a necessary and important step toward public health policy making and raising public awareness. Our study provides a comprehensive snapshot of dietary correlates of IR and inflammation markers at a national level and, given the nature of this study, it could be extrapolated to the US adult population.

## Conclusions

In conclusion, our findings, based on a large and representative sample size, provide insights into the protective role of WG intake on IR and inflammatory markers, which may be mediated, at least partially, by adiposity. This finding may have implications on health policies in relation to dietary strategies to prevent and control obesity-related conditions involving IR or systemic inflammation.

## Abbreviations

apo-B: Apolipoprotein B
BMI: Body mass index
CHD: Coronary heart disease
CRP: C-reactive protein
CVD: Cardiovascular disease
DM: Diabetes mellitus
FBG: Fasting blood glucose
HbA_1c_: Hemoglobin A_1c_
HDL-C: High density lipoprotein-cholesterol
HOMA-IR: Homeostatic model assessment of IR
HOMA-β: β cell function
IR: Insulin resistance
NCHS: National Center for Health Statistics
NHANES: National Health and Nutrition Examination Survey
RCT: Randomized control trials
TyG: Triglyceride-glucose
WG: Whole grain
